# Exposure to Brominated Trihalomethanes in Water During Pregnancy and Micronuclei Frequency in Maternal and Cord Blood Lymphocytes

**DOI:** 10.1289/ehp.1206434

**Published:** 2013-11-01

**Authors:** Leslie Thomas Stayner, Marie Pedersen, Evridiki Patelarou, Ilse Decordier, Kim Vande Loock, Leda Chatzi, Ana Espinosa, Eleni Fthenou, Mark J. Nieuwenhuijsen, Esther Gracia-Lavedan, Euripides G. Stephanou, Micheline Kirsch-Volders, Manolis Kogevinas

**Affiliations:** 1 Division of Epidemiology and Biostatistics, School of Public Health, University of Illinois at Chicago, Chicago, Illinois, United States; 2 Centre for Research in Environmental Epidemiology (CREAL), Barcelona, Spain; 3 CIBER Epidemiologia y Salud Pública (CIBERESP), Madrid, Spain; 4 INSERM (National Institute of Health and Medical Research), U823, Team of Environmental Epidemiology Applied to Reproduction and Respiratory Health, Institute Albert Bonniot, Grenoble, France; 5 Department of Postgraduate Research, Florence Nightingale School of Nursing and Midwifery, King’s College London, London, United Kingdom; 6 Department of Social Medicine, Medical School, University of Crete, Heraklion, Crete, Greece; 7 Laboratory of Cell Genetics, Faculty of Science and Bio-engineering, Vrije Universitei Brussel, Brussels, Belgium; 8 IMIM (Hospital del Mar Medical Research Institute), Barcelona, Spain; 9 Environmental Chemical Processes Laboratory, Department of Chemistry, University of Crete, Heraklion, Crete, Greece; 10 National School of Public Health, Athens, Greece

## Abstract

Background: Water disinfection by-products have been associated with an increased cancer risk. Micronuclei (MN) frequency in lymphocytes is a marker of genomic damage and can predict adult cancer risk.

Objective: We evaluated maternal exposure to drinking water brominated trihalomethanes (BTHM) in relation to MN frequency in maternal and cord blood lymphocytes.

Methods: MN frequency was examined in 214 mothers and 223 newborns from the Rhea mother–child cohort in Crete, Greece, in 2007–2008. Residential BTHM water concentrations were estimated during pregnancy using tap water analyses and modeling. Questionnaires on water related habits were used to estimate BTHM exposure from all routes. Associations between BTHM and MN frequency were estimated using negative binomial regression.

Results: BTHM concentrations in residential tap water during pregnancy ranged from 0.06 to 7.1 μg/L. MN frequency in maternal binucleated lymphocytes was found to increase with BTHM concentrations in residential water for exposure during the first [rate ratio (RR) for 1 μg/L = 1.05; 95% CI: 1.00, 1.11] and second trimesters (RR for 1 μg/L = 1.03; 95% CI: 1.00, 1.06), and through all routes of BTHM exposure during the first trimester (RR for 1 μg/week = 3.14; 95% CI: 1.16, 8.50).

Conclusions: These findings suggest that exposure to BTHM may increase the frequency of MN in maternal binucleated lymphocytes.

Citation: Stayner LT, Pedersen M, Patelarou E, Decordier I, Vande Loock K, Chatzi L, Espinosa A, Fthenou E, Nieuwenhuijsen MJ, Gracia-Lavedan E, Stephanou EG, Kirsch-Volders M, Kogevinas M. 2014. Exposure to brominated trihalomethanes in water during pregnancy and micronuclei frequency in maternal and cord blood lymphocytes. Environ Health Perspect 122:100–106; http://dx.doi.org/10.1289/ehp.1206434

## Introduction

Disinfection of water reduces pathogenic infection; however, water disinfectants also react with organic materials to generate disinfection by-products (DBP). Several hundred DBPs have been identified. Trihalomethanes (THM) are one of the most frequent classes of DBP detected in residential water (Richardson et al. 2007).

Several DBP compounds, such as brominated THM (BTHM), are carcinogenic in rodents and are mutagenic in several tests [International Agency for Research on Cancer (IARC) 2004; Richardson et al. 2007]. There is epidemiologic evidence of an association between bladder cancer and exposure to DBP from residential water (Villanueva et al. 2004) and from bathing, showering, or swimming (Villanueva et al. 2006).

Micronuclei (MN) frequency is a well-established biomarker of DNA damage (Fenech 2007). MN frequency in peripheral blood lymphocytes has been associated in a large prospective study with an increased risk of cancer among adults (Bonassi et al. 2011).

The frequency of MN has been reported to increase in exfoliated urine cells with THM exposures (Villaneuva et al. 2006). BTHM concentrations in exhaled breath have been associated with an increased frequency of MN in lymphocytes and increased urinary mutagenicity in adults swimming in indoor pools (Kogevinas et al. 2010).

Little is known about potential mutagenic or carcinogenic effects of *in utero* exposures to DBP. The fetus may be more susceptible to environmental mutagens and carcinogens (Wild and Kleinjans 2003), and certain cancers may have a fetal origin (Gluckman et al. 2008). With this in mind, we assessed the potential associations between exposure to BTHM during pregnancy and MN frequency in maternal and cord blood lymphocytes.

## Methods

*Study population*. This study includes mothers and newborns from the island of Crete, Greece, who were part of the Rhea cohort (Chatzi et al. 2009; Patelarou et al. 2011). Women who became pregnant during February 2007–February 2008 were asked to participate in the study. The first contact with potential participants was made at around 10–13 weeks of pregnancy, at the time of the first ultrasound examination. Women were invited to provide blood and urine samples and complete face-to-face interviews if they were residents of the study area, > 16 years of age, visiting a participating hospital or private clinic during the 10th–13th week of gestation, and did not have communication limitations. Participating women were contacted again during the 14th–18th and 28th–32nd weeks of pregnancy and at birth. During recruitment, 1,610 eligible women agreed to participate, and 1,459 (91%) were followed through delivery. A subset of 408 participants donated maternal and/or cord blood for biomarker measurements as part of the NewGeneris study (Merlo et al. 2009). A total of 214 mothers and 223 newborns with MN analysis of maternal and cord blood lymphocytes from singleton pregnancies were included in this study. Of these, 162 were mother–child pairs.

A comparison of those who did and did not participate in the current study revealed a small but significantly lower percentage of Greek women among those who participated in the study (87%) than among those who did not (92%). Otherwise the groups were very similar with regard to the study covariates (data not shown). Questionnaires elicited information on maternal characteristics including ethnicity, age, prepregnancy body mass index (BMI), parity (none or ≥ 1), smoking, secondhand tobacco smoke (SHS), supplement use, education, and water-related habits during pregnancy (Patelarou et al. 2011). A food frequency questionnaire was used to assess dietary habits and adherence to a Mediterranean diet (Chatzi et al. 2012). Information on diabetes, gestational hypertension, and anthropometric measurements at birth was obtained from medical records. Gestational age was based on the interval between the last menstrual period and the date of delivery for 84% of the women. When the last menstrual estimate was inconsistent by ≥ 7 days with the ultrasound measurement taken in the first trimester of pregnancy (16%), a quadratic regression formula describing the relation between crown–rump length and gestational age was used (Westerway et al. 2000).

The Ethics Committee of the University Hospital in Heraklion, Crete, Greece, approved the study. All participants provided written, informed consent for themselves and their child after having received a complete description of the study.

*DBP exposure assessment*. As part of the European project HIWATE (Health Impacts of long-term exposure to disinfection byproducts in drinking Water), residential and maternal exposure to DBP during pregnancy was estimated using standardized methods (Nieuwenhuijsen et al. 2009; Patelarou et al. 2011). Briefly, on the basis of information provided by the water company that supplies Heraklion, the city was divided into six zones according to the source of underground water used in each area that corresponded to the six water treatment plants supplying water. In total, 18 sampling points were selected (12 areas in Heraklion and 6 in rural areas), which covered geographically the residences of participating women. One household in each of the 18 areas, which was randomly selected, was visited four times at home to collect tap water samples between the months of September 2007 and January 2009 (72 tap water samples in total). Samples were analyzed by gas chromatography/mass spectrometry for the four specific THMs: chloroform, bromoform, bromodichloromethane, and dibromochloromethane. BTHM constituted > 80% of the total THM (Patelarou et al. 2011), so we decided to concentrate this analysis on exposures to BTHM. Residential addresses were assigned to a water supply zone based on the maternal address early in pregnancy. BTHM concentrations in residential water were estimated based on the residential water supply zone, and individual residential concentrations of BTHM (micrograms per liter of residential water) were predicted using generalized additive models for each month of pregnancy.

Average residential water BTHM concentrations were calculated for the entire pregnancy and for each trimester for both mothers and newborns. BTHM concentrations were assumed to be uniform within the water supply zones, so the potential spatial variation of BTHM concentrations within the water systems was not considered in the analysis. However, the areas covered by these systems were small, and little variation within the systems was expected (Symanski et al. 2004).

Information on maternal water usage habits during pregnancy was combined with estimated residential water concentrations to estimate maternal and prenatal BTHM exposures through all routes, including ingestion, dermal absorption, and inhalation (micrograms per week) (Patelarou et al. 2011).

*MN frequency in maternal and cord blood lymphocytes*. Maternal peripheral blood was collected by venipuncture 1 day after delivery, and cord blood was collected by umbilical puncture from the placenta immediately after delivery (Merlo et al. 2009). The blood for the cytokinesis-block micronuclei assay was kept in heparin tubes at 4°C. Whole-blood cultures with phytohemagglutinin (PHA) stimulation were initiated within 24 hr after collection (Vande Loock et al. 2011). After 44 hr of incubation, cytochalasin B was added to the cultures to block the cell division and enable scoring of binucleated (BN; i.e., cells that have divided once, having two nuclei) lymphocytes. At 72 hr following PHA stimulation, the cultures were harvested. A hypotonic treatment was used to remove the red blood cells; cells were fixed in freshly prepared methanol-acetic acid, and cell suspensions were dropped onto clean slides. Slides were stained and were examined in the Laboratory of Cell Genetics, Vrije Universiteit Brussel.

A semi-automated scoring procedure of the lymphocytes was carried out, using the PathFinder platform installed by Imstar (version 6; Paris, France). At the end of the automatic screening, lymphocytes containing detected MN were visually evaluated. After validation, the total numbers of BN and mononucleated (MONO) lymphocytes with MN, and the total number of lymphocytes counted for each participant, were recorded. The cytokinesis-block proliferation index (CBPI) for each participant was calculated as follows: CBPI = (number of MONO + 2 × number of BN + 3 × number of polynucleated lymphocytes)/total number of lymphocytes counted (Decordier et al. 2009). The design of the MN protocol aims at maximizing the number of BN lymphocytes, which results on average in a CBPI of 2. Lower CBPI frequencies reflect cell cycle delay due to toxicity or cell immaturity, and higher CBPI frequencies result from excessive mitogenic activation.

*Statistical analyses*. Descriptive analyses of the study population characteristics, exposures, and outcomes were conducted. Negative binomial regression was used to model the MN counts using the GLM (generalized linear models) procedure in STATA 11.0 and 13.0 (StataCorp, College Station, TX, USA). A natural log link was used, and the model included the number of either BN or MONO cells as an offset to control for the number of lymphocytes counted. We also evaluated Poisson regression as an alternative to negative binomial regression, but the latter provided a better fit to the data (data not shown). MN frequencies in maternal BN and MONO, and cord blood BN and MONO, were evaluated as outcomes using separate models to estimate associations of each outcome with BTHM concentrations in residential water (micrograms per liter; categorized into quartiles), and BTHM exposures through all routes (micrograms per week and as quartiles), for each trimester and for the entire pregnancy. In addition, we used separate models to estimate associations of the outcomes with maternal water habits during early pregnancy, including bathing and showering [evaluated separately according to frequency (times per week), duration (minutes), or frequency × duration (minutes/week); and as shower only, bath only, or both], use of a swimming pool during pregnancy (any or none), hand dishwashing only (vs. any use of a dishwashing machine), and type of drinking water consumed (public, bottled, or spring water). Models were also fitted to evaluate the possible effect of clustering by the water systems by including a random effect for water systems using the VCE (variance covariance estimation) option in the STATA GLM procedure.

Covariates for control of potential confounding included a set of variables that were believed *a priori* to be determinants of MN. For mothers, the models included maternal age at birth (years), smoking during pregnancy (nonsmoker/ex-smoker/active smoker), workplace and home exposure to SHS (no/yes), residence in an urban area (no/yes), education (low: ≤ 9 years education; medium: 10–14 years education; high: university degree or higher), and ethnicity (Greek/non-Greek). For newborns, the same covariates were included, except that gestational age (weeks) was substituted for maternal age. Nonsmokers reported not smoking at any time for at least 3 months before pregnancy or during pregnancy; ex-smokers reported smoking sometime within the 3 months before pregnancy and/or during the first 12 weeks of gestation, but not after then; and active smokers reported smoking 3 months before pregnancy and at approximately 12 weeks of gestation (the time of interview). In addition, potential confounding by prepregnancy maternal BMI (kilograms per meter squared), prior births (yes/no), Mediterranean diet (low, moderate, high), use of dietary supplements (yes/no), diabetes (yes/no), gestational hypertension (yes/no), and small for gestational age (yes/no) was evaluated by fitting models with and without these covariates and monitoring changes in the regression coefficient of the exposure variables. Statistical testing and 95% CIs were estimated using robust standard errors. Restricted cubic spline models with 3 knots were fitted based on Harrell’s recommended percentiles to evaluate the linearity of associations. Statistical interactions between the exposure variables and all of the covariates were assessed by fitting models with cross-product terms and testing their significance using a Wald test. The cross-product terms corresponding to categorical covariates with > 2 levels were tested as a group. Any interactions that were found to be statistically significant at the *p* < 0.05 level were included in the models.

The inclusion of numerous potential confounders in the analysis resulted in a large amount of missing data. To increase efficiency and reduce selection bias, we applied multiple imputation methods. The imputation used all the available information on the individual characteristics of the entire study population and included all individuals who had MN data for either the mother or the child. To make the missing-at-random (MAR) assumption more plausible, we included many variables in the imputation model and used the chained equations procedure (White et al. 2011) under the MAR assumption. Categorical variables were modeled using logistic or ordinal models; non-normally distributed variables were log-transformed or imputed using predictive mean matching. All of the covariates were included in the model. We generated 30 complete data sets that were analyzed individually to obtain a set of parameters and then combined into overall estimates using Rubin’s rule (Rubin 1987).

## Results

*Study population characteristics*. A summary of the variables used in the analysis for the combined population (*n* = 240) of mothers and newborns with complete MN and BTHM information for all and stratified by quartiles of BTHM exposure averaged over the pregnancy is presented in Table 1. Most of the mothers were Greek (86%) and ≤ 35 years of age (88%). Most mothers did not smoke during pregnancy (61%), but were exposed to SHS either at home or work (89%), had moderate education (53%), and lived in urban areas (72%). Most women drank bottled water (73%) and only used showers (vs. baths) (95%) during pregnancy. Six percent of the children were born before 37 weeks of gestation. The mean (± SD) gestational age was 38.4 ± 1.3 weeks, and mean birth weight was 3,225 ± 412 g.

**Table 1:** Study population characteristics [*n* (%)]^*a*^ for all and by quartiles of the residential exposure to brominated trihalomethanes during the full pregnancy.^*b*^

Characteristic	All (*n* = 240)	Quartile 1	Quartile 2	Quartile 3	Quartile 4	*p*-Value^*c*^
Ethnicity						0.82
Greek	205 (86.3)	60 (88.2)	43 (84.3)	50 (83.3)	30 (88.1)
Other	33(13.9)	8 (11.8)	8 (15.7)	10 (16.7)	7 (11.9)
Missing	2	1	0	0	1
Maternal age (years)						0.24
≤ 35	210 (87.8)	65 (94.2)	44 (88.0)	50 (83.3)	51 (85.0)
> 35	29 (12.1)	4 (5.8)	6 (12.0)	10 (16.7)	9 (15.0)
Missing	1	0	1	0	0
Maternal smoking						0.06
Nonsmokers	145 (61.2)	45 (65.2)	33 (68.9)	28 (46.7)	39 (65.0)
Ex-smokers	44 (18.6)	16 (23.2)	5 (10.4)	14 (23.3)	9 (15.0)
Active smokers	48 (20.2)	8 (11.6)	10 (20.8)	18 (30.0)	12 (20.0)
Missing	3	0	3	0	3
SHS^*d*^						0.42
Not exposed	26 (10.9)	5 (7.3)	5 (10.0)	6 (10.0)	10 (16.7)
Exposed	213 (89.1)	64 (92.8)	45 (90.0)	54 (90.0)	50 (83.3)
Missing	1	0	1	0	0
Maternal education						0.68
Low	55 (23.0)	19 (27.5)	11 (22.0)	13 (21.7)	12 (20.0)
Moderate	126 (52.7)	36 (52.2)	30 (60.0)	30 (50.0)	30 (50.0)
High	58 (24.3)	14 (20.3)	9 (18.0)	17 (28.3)	18 (30.0)
Missing	1	0	0	0	0
Residence						< 0.001
Urban	171 (72.1)	38 (55.9)	26 (52.0)	55 (91.7)	52 (88.1)
Rural	66 (27.9)	30 (44.1)	24 (48.0)	5 (8.3)	7 (11.9)
Missing	3	1	1	0	0
Gestational age (weeks)						0.98
≥37	225 (93.8)	64 (92.8)	48 (94.1)	57 (95.0)	56 (93.3)
< 37	15 (6.3)	5 (7.3)	3 (5.9)	3 (5.0)	4 (6.7)
Missing	0	0	0	0	0
Drinking water						< 0.001
Bottled	174 (72.8)	40 (58.0)	31 (62.0)	53 (88.3)	50 (83.3)
Public	49 (20.5)	23 (33.3)	15 (30.0)	6 (10.0)	5 (8.3)
Spring	16 (6.7)	6 (8.7)	4 (8.0)	1 (1.7)	5 (8.3)
Missing	1	0	1	0	0
Type of bathing						0.91
Shower only	221 (94.5)	63 (94.0)	48 (98.0)	55 (84.8)	55 (91.7)
Bath only	5 (2.1)	2 (3.0)	0 (0.0)	1 (1.7)	2 (3.3)
Both	8 (3.4)	2 (3.0)	1 (2.0)	2 (3.5)	3 (5.0)
Missing	6	2	2	2	0
Swimming pool use						0.24
Ever	229 (98.7)	67 (100.0)	47 (97.9)	57 (100.0)	58 (96.7)
Never	3 (1.3)	0 (0.0)	1 (2.1)	0 (0.0)	2 (3.3)
Missing	8	2	3	3	0
Hand dishwashing						0.21
No	82 (35.2)	25 (37.3)	13 (27.1)	26 (44.8)	18 (30.0)
Yes	151 (64.8)	42 (62.7)	35 (72.9)	32 (55.2)	42 (70.0)
Missing	7	2	3	2	0
All variables correspond to maternal characteristics during pregnancy. ^***a***^Number of subjects with micronuclei analyses (either mother or newborn). ^***b***^Brominated trihalomethanes in residential water quartiles: 0.06–0.15; > 0.15–0.74; > 0.74–4.44; > 4.44–7.09 μg/L. ^***c***^*p*-Value based on Fisher exact test for table ­frequencies (excluding the missing values). ^***d***^Exposed to secondhand smoke either at work or at home.						

All of the study covariates had missing data for some participants except sex and gestational age. The percentage of missing values ranged from 0.4% (delivery mode) to 31% (Mediterranean diet). The percentage of participants missing data for at least one of the final model covariates was 3%.

Maternal residence in an urban area (*p* < 0.001), and drinking-water source (*p* < 0.001) were the only covariates that were significantly associated with BHTM (Table 1). The percentage of women who were in the highest quartile of BTHM was higher for those living in an urban area (27%) than those living in a rural area (10%). The percentage of women in the highest quartile of BTHM who consuming bottled water only (28%) was greater than those who consumed public (9%) or spring (4%) water.

*Residential exposure to BTHMs during pregnancy*. For the entire pregnancy, the mean concentration of BTHM in residential water was 2.1 ± 2.6 μg/L and the median was 0.8 μg/L. The minimum residential concentration was 0.06 μg/L and the maximum was 7.1 μg/L. Average BTHM concentrations over pregnancy were strongly correlated for the first and second trimester [Pearson correlation coefficient (*r*) = 0.70] and the second and third trimester (*r* = 0.73), but the correlation between the first and third trimester was weaker (*r* = 0.31). The mean maternal exposure through all routes was estimated to be 0.06 ± 0.10 μg/week. There was a strong positive correlation between mean residential water BTHM concentrations and estimated maternal BTHM exposure through all routes (*r* = 0.74, *p* < 0.001). Data for residential water concentrations and all route exposure estimates were missing and imputed for 25 (12%) mothers and 28 (13%) newborns.

*MN frequency distribution*. The mean MN frequency per 1,000 BN was 2.85 ± 2.20 (range, 0.00–15.88) in the 214 maternal blood samples and 1.80 ± 1.51 (range, 0.00–8.47) in the 223 cord blood samples (*p* < 0.001, paired *t*-test comparing mother and newborn counts based on the 162 paired samples). The mean frequency of MN per 1,000 MONO was 0.71 ± 1.04 (range, 0.00–6.79) for the mothers and 0.62 ± 0.72 (range, 0.00–4.19) for the newborns (*p* = 0.37, paired *t*-test for mother–newborn comparison). The mean CBPI was 1.64 ± 0.25 (range, 1.08–2.16) for maternal cultures and 1.57 ± 0.20 (range, 1.03–1.98) for the cord blood cultures (*p* = 0.99, for paired *t*-test for mother–newborn comparison). A trend of increasing mean MN frequencies with increasing pregnancy mean BHTM exposures was observed for both BN (*p* = 0.07) and MONO (*p* = 0.02) in mothers only (see Supplemental Material, Table S1).

*Regression model findings*. Compared with taking showers only, taking baths only was associated with an increased frequency of MN in maternal BN [rate ratio (RR) = 2.08; 95% CI: 1.09, 3.98] and MN in maternal MONO (RR = 6.72; 95% CI: 3.21, 14.05) based on five mothers who only used a bath (Table 2). The frequency of MN in maternal MONO also increased with the number of baths per week (RR = 1.30; 95% CI: 1.08, 1.55 per bath), and with the product of duration and the frequency of bathing per week (RR = 1.65; 95% CI: 1.23, 2.21 for 60 min of bathing per week). Conversely, the frequency of MN in maternal MONO decreased with frequent showering (RR = 0.88; 95% CI: 0.82, 0.95 for each additional shower per week) and with the product of duration and the frequency of showering per week (RR = 0.72; 95% CI: 0.52, 1.00 for 60 min of showering per week). Similar findings were observed for the analyses of the MN in maternal BN for bathing and showering duration and times per week except that these findings were not statistically significant. Compared with public water as a drinking source, maternal consumption of spring water only was associated with decreasing MN frequency in maternal MONO lymphocytes (RR = 0.26; 95% CI: 0.10, 0.70, based on 16 women who used spring water), and nonsignificantly related to decreased MN in maternal BN and MONO in newborns.

**Table 2:** Associations between maternal water usage habits and micronuclei frequency per 1,000 BN and MONO lymphocytes from maternal blood and newborns cord blood samples [RR (95% CI)].

Variable		Maternal blood cells^*a*^		Newborns cord blood cells^*b*^	
		BN	MONO	BN	MONO
Type of bathing	Showers only	1.00	1.00	1.00	1.00
	Baths only	2.08 (1.09, 3.98)*	6.72 (3.21, 14.1)^#^	1.07 (0.80, 1.42)	1.13 (0.62, 2.07)
	Both	0.79 (0.54, 1.16)	0.20 (0.04, 1.05)	1.17 (0.70, 1.78)	1.13 (0.59, 2.14)
Showering	Times per week	0.98 (0.95, 1.00)	0.88 (0.82, 0.95)**	0.98 (0.96, 1.01)	1.03 (0.99, 1.07)
	Duration^*c*^	0.96 (0.86, 1.08)	0.86 (0.66, 1.12)	0.86 (0.75, 1.00)*	0.93 (0.77, 1.13)
	Times × duration^*d*^	0.95 (0.87, 1.05)	0.72 (0.52, 1.00)**	0.84 (0.73, 0.97)*	1.06 (0.91, 1.24)
Bathing	Times per week	1.09 (0.97, 1.23)	1.30 (1.08, 1.55)**	1.09 (0.99, 1.19)	1.06 (0.95, 1.18)
	Duration^*c*^	1.06 (0.92, 1.23)	1.30 (0.97, 1.74)	1.07 (0.93, 1.22)	1.02 (0.82, 1.26)
	Times × duration^*d*^	1.19 (0.96, 1.47)	1.65 (1.23, 2.21)**	1.15 (0.92, 1.44)	1.15 (0.90, 1.48)
Swimming pool use^*e*^		0.88 (0.32, 2.42)	1.19 (0.49, 2.88)	1.08 (0.32, 3.66)	1.74 (0.29, 10.41)
Drinking water source	Public water	1.00	1.00	1.00	1.00
	Bottled	0.88 (0.68, 1.13)	0.75 (0.43, 1.33)	1.12 (0.82, 1.52)	0.78 (0.55, 1.09)
	Spring	0.79 (0.54, 1.16)	0.26 (0.10, 0.70)**	1.09 (0.68, 1.74)	0.75 (0.37, 1.51)
Hand dishwashing^*f*^		1.11 (0.89, 1.37)	0.76 (0.44, 1.34)	0.89 (0.70, 1.15)	0.89 (0.66, 1.21)
RRs and 95% CIs of the micronuclei frequency from negative binomial models. ^***a***^Models for mothers controlled for maternal age at birth (years), maternal active smoking (nonsmokers/ex-smokers/active smokers), workplace and home SHS (no/yes), living in an urban area (no/yes), maternal education (low/middle/high), and maternal Greek ethnicity (yes/no). ^***b***^Models for newborns controlled for the same variables as the maternal models except that maternal age was replaced by gestational age (weeks). ^***c***^Results presented are for 10 min of showering or bathing. ^***d***^Results presented are for 60 min of showering or bathing per week. ^***e***^Results presented are for comparison of never (reference group) and ever used swimming pool during pregnancy. ^***f***^Results presented are for comparison of dishwashing by machine (reference group) and hand. *p < 0.05. **p < 0.01. ^#^*p *< 0.001.					

The results from modeling the frequency of MN in BN in mothers and newborns using continuous exposure variables are presented in Table 3. Associations were closer to the null when adjusted for covariates compared with unadjusted estimates (see Supplemental Material, Tables S2–S4). The frequency of MN in maternal BN increased with BTHM concentrations in residential water during the first and second trimesters (RR = 1.05; 95% CI: 1.00, 1.11 *p* = 0.06 and RR = 1.03; 95% CI: 1.00, 1.06 *p* = 0.04 per 1-μg/L increase in BTHM, respectively) and with the average concentration during pregnancy (RR = 1.03; 95% CI: 0.99, 1.07, *p* = 0.13). When residential BTHM concentrations were categorized into quartiles (data not shown), adjusted RRs for the frequency of MN in maternal BN in the highest quartile relative to the first quartile were 1.28 (95% CI: 0.97, 1.70) and 1.24 (95% CI: 0.95, 1.61) for concentrations during the first and second trimesters, respectively. However, residential BTHM concentrations during the third trimester were not associated with MN in maternal BN (*p* = 0.95).

**Table 3:** Associations between brominated trihalomethanes and micronuclei frequency in BN cells from maternal and cord blood samples [RR (95% CI)].

Exposure	Mothers^*a*^	Newborns^*b*^
Residential tap water (μg/L)
1st trimester	1.05 (1.00, 1.11)	1.02 (0.95, 1.08)
2nd trimester	1.03 (1.00, 1.06)	0.99 (0.96, 1.02)
3rd trimester	1.00 (0.98, 1.03)	0.98 (0.95, 1.01)
Full pregnancy	1.03 (0.99, 1.07)	0.98 (0.94, 1.03)
All routes of exposure (μg/week)
1st trimester	3.14 (1.16, 8.50)	0.90 (0.23, 1.48)
2nd trimester	1.68 (0.76, 3.73)	0.56 (0.26, 1.21)
3rd trimester	0.76 (0.40, 1.45)	0.59 (0.28, 1.24)
Full pregnancy	1.55 (0.59, 4.09)	0.51 (0.19, 1.36)
RRs and 95% CI of the micronuclei frequency in maternal mononucleated cells from negative binomial models (per 1 unit of exposure increment). ^***a***^Adjusted for maternal age at birth, maternal tobacco smoking, maternal exposure to workplace and home SHS, living in an urban area, maternal education, and Greek ethnicity. ^***b***^Models for newborns controlled for the same variables as the maternal models except that maternal age was substituted by gestational age at birth (weeks).		

A 1-μg/week increase in BTHM exposures from all routes during the first trimester also was associated with higher frequency of MN in maternal BN (RR = 3.14; 95% CI: 1.16, 8.50 *p* = 0.03) (Table 3). The association with BTHM exposures from all routes during the second trimester was also associated with elevated MN frequency in maternal BN, but this association was statistically nonsignificant (RR = 1.68; 95% CI: 0.76, 3.73 *p* = 0.2).

Plots of the model-predicted exposure–response relationships between residential BTHM water concentrations and the frequency of MN in maternal BN from unadjusted models are presented in the Supplemental Material, Figure S1. A restricted cubic spline with 3 knots is also included in the plots as a means of assessing the linearity of the exposure–response trends. These plots indicate that the linear models provide a reasonable fit to the estimates from the categorical and cubic spline models. Similar trends were evident when models with adjustment for the *a priori* covariates (results not shown).

There was no evidence of associations between any of the BTHM exposure measures and the frequency of MN in cord blood BN (see Supplemental Material, Table S2). For both mothers and newborns, there was no evidence of an association between any of the BTHM exposure estimates and the MN frequency in MONO (see Supplemental Material, Table S3 and Table S4).

Inclusion of additional covariates had little influence on model estimates (data not shown). Estimates were comparable to those shown when the analysis was restricted to the subset of study population with complete data (i.e., without the use of multiple imputation to impute missing data) (data not shown).

Mixed models that included a random effect for water systems had no influence on the exposure regression coefficients, but increased the precision and reduced the *p*-values for the estimated effect of BTHM exposures during the first trimester for residential exposures (from *p* = 0.03 to *p* = 0.01) and for all routes of exposure (from *p* = 0.01 to *p* < 0.001). Only minor changes were observed in the results for the other BTHM regression parameters.

A statistically significant interaction was observed between maternal smoking (nonsmokers/ex-smokers/active smokers) and BHTM exposures experienced during the first trimester for both residential water concentrations (*p* < 0.001) and all routes of exposure (*p* = 0.02) for MN frequency in maternal BN. Stratified analyses revealed that BHTM exposures during the first trimester were not associated with MN in maternal BN among the 145 nonsmokers for either residential water (RR = 0.99; 95% CI: 0.93, 1.04) or for all routes of BHTM exposure (RR = 1.23; 95% CI: 0.34, 4.50) (see Supplemental Material, Table S5). A highly significant (*p* = 0.002) association was estimated for residential BHTM contaminants among the 48 active smokers (RR = 1.11; 95% CI: 1.04, 1.18). A borderline significant (*p* = 0.07) association was estimated for all routes of exposure among the 48 active smokers (RR = 3.97; 95% CI: 0.88, 17.99) and for residential water contaminants among the 44 ex-smokers (RR = 1.18; 95% CI: 0.99, 1.42).

Statistically significant (*p* < 0.01) interactions were also observed in the models for MN in maternal BN between maternal education and the all routes of exposure variables for second and third trimesters, and for the average over the pregnancy. The interaction for maternal education and all routes of exposure during the first trimester was nearly statistically significant (*p* = 0.10). Stratified analyses indicated that positive associations with all routes of BHTM exposure were only evident among the 47 women with low education (1st trimester: RR = 18.09; 95% CI: 1.31, 250.04; 2nd trimester: RR = 7.98; 95% CI: 1.17, 37.17; 3rd trimester: RR = 1.59; 95% CI: 0.55, 4.63; and pregnancy average: RR = 10.88; 95% CI: 1.70, 69.48) (see Supplemental Material, Table S6).

## Discussion

To our knowledge, the present study is the first to examine the relationship between exposure to BTHM and MN frequency in maternal and cord blood lymphocytes. We found evidence of a significant increase of MN frequency in maternal BN with BTHM exposures during the first and second trimesters. An association between BTHM exposures during the third trimester and maternal MN frequency in BN was not evident. There was also no evidence of BTHM exposures being associated with either maternal MN frequency in MONO or MN frequency in newborns.

Our finding of an association between exposure to BTHMs and higher MN frequency in maternal BN lymphocytes are consistent with two other epidemiologic studies. Villanueva et al. (2006) reported an increase in the frequency of MN in exfoliated urine cells with higher BTHM exposures among adults. Kogevinas et al. (2010) also reported an association between BTHM in exhaled breath and an increased frequency of MN in BN lymphocytes in adults following swimming in an indoor pool. However, a third study conducted in Australia did not find an association between THM in drinking water and MN frequency in urothelial cells from adults (Ranmuthugala et al., 2003).

There is some support for the biologic plausibility of our findings of an association from experimental studies. BTHM have been found to be genotoxic in experimental systems (Le Curieux et al. 1995). Brominated DBPs were reported to be more mutagenic and cytotoxic than chlorinated DBPs based on *in vitro* assays of Chinese hamster ovary cells (Plewa et al. 2010). Experimental studies based on animal models or *in vitro* assays have provided inconsistent findings regarding the effects of brominated compounds on MN frequency. MN in cells exposed *in vitro* increased linearly with the concentration of bromide ions (Nobukawa and Sanukida 2001). MN frequency in the mouse bone marrow was not found to increase following a single intraperitoneal injection of bromodichloromethane and dibromochloromethane (Hayashi et al. 1988). Intraperitoneal injection of bromoform induced MN in one study (Melnick 1989), but not by gavage in another (Stocker et al. 1997). MN induction was observed in p53 (±) transgenic mice exposed by inhalation, but only at high concentrations (15 ppm) relative to environmental levels (Torti et al. 2002). The tissue, route, and short duration of exposure in these studies make them of questionable value for evaluating the risk of MN formation in humans following chronic exposures to mixtures of BTHM by multiple routes.

Of course it is possible that our positive findings might be the result of unrecognized confounding. There are no obvious industrial sources of pollution in the residential areas that were included in this study. Age has been strongly associated with higher MN frequency in adults, particularly among women, which was also true in our models. Tobacco smoke and air pollution have also been associated with higher MN frequency (Pedersen et al. 2009; Rossnerova et al. 2011; Zalacain et al. 2006). Our findings are adjusted for both active smoking and SHS, and for living in an urban or rural area. It is possible that residual confounding may exist by these factors, because we did not have the level of smoking, and residing in an urban area is a crude proxy for air pollution. In Heraklion, phthalates have been detected in water storage tanks that are used during periods of water shortages. However, insufficient information is available to quantify the potential for exposure to phthalates from these tanks.

We are not aware of a biologic mechanism that would explain stronger associations with exposures during the first trimester than exposures during other trimesters. There was no *a priori* time period during which we expected a maximum effect, and we estimated effects by trimester primarily for the newborns. A possible explanation is that the exposure estimates were based on the residence around the third month of pregnancy; thus, the potential for exposure misclassification was the smallest during the first trimester. However, residential mobility among pregnant women was very low in this population (9%) (Patelarou et al. 2011).

The association of BTHM with MN in maternal BN was modified by smoking and maternal education. Significant positive associations were estimated only among women who smoked during their pregnancy, and among women with less education. The interaction with education may be related to factors not assessed in this study, such as exposure to ambient air pollution, a maternal diet high in genotoxic compounds, and/or low in antioxidants, but might also simply be a chance finding related to the multiple comparisons performed in this study. There is limited biologic support in the literature for an interaction with smoking. Smoking has been shown to increase expression of glutathione *S*-transferase (GST) (Thum et al. 2006), which is a key metabolic pathway for the biotransformation and genotoxicity of BTHM (DeMarini et al. 1997; Pegram et al. 1997).

There was no evidence in our study that exposure to BTHM had any effect on the MN frequency in lymphocytes from cord blood. It has been shown that small THM molecules, such as chloroform, can pass from the maternal circulation to the fetal circulation through the placenta (Dowty et al. 1976). The lack of associations between exposures and MN in newborns might be explained by the fact that the maternal–placental–fetal circulation becomes established only around the 10th week of pregnancy, whereas the majority of lymphocytes collected at birth in cord blood are produced during the third trimester (Blackburn 2007). If the critical window of exposure was during the first trimester, then the cord blood lymphocytes collected at birth may not have been exposed. Another possible biologic explanation is that certain BTHM are metabolized by GST to reactive mutagens (DeMarini et al. 1997; Pegram et al. 1997), and it is likely that *in utero* metabolism of BTHM is immature, resulting in a lower exposure of the reactive toxic metabolites of THM to the fetus (Wild and Kleinjans 2003).

Exposure to BTHM was not associated with higher MN frequencies in MONO in either mothers or newborns. This might be explained by the fact that MN in MONO is less common than MN in BN, resulting in lower statistical power. Another explanation is that MN frequencies in MONO are thought to provide an estimate of genomic instability accumulated over many years in stem cells and circulating T lymphocytes, whereas MN in BN provide a measure of the lesions that have accumulated in the DNA or in key proteins since the lymphocytes last replicated *in vivo* (Kirsch-Volders and Fenech 2001; Kirsch-Volders et al. 2011). Thus, the estimates of BTHM exposures over the course of the pregnancy may not be relevant for studying MN frequencies in MONO for the mothers, which may have been influenced by earlier exposures.

Taking baths was strongly associated with MN frequency in maternal BN and MONO lymphocytes based on a small number of women who did not use showers (*n* = 5). Bathing has been reported to result in a higher uptake of THM through both skin absorption and inhalation than showering (Gordon et al. 1998). Furthermore, in the present study population, the duration of bathing was on average longer than the time showering (32 vs. 14 min), increasing the potential for exposure to BTHM. Compared with the 43 women who consumed public water, the 11 women who consumed only spring water had a reduced frequency of MN in MONO lymphocytes. Spring water is generally not chlorinated, so women consuming spring water would have low exposures to THM in their drinking water.

This study had a number of strengths and some weaknesses as well. The strengths include the fact that MN frequency was available from a relatively large and well-characterized sample of maternal and cord blood, and that BTHM concentrations were estimated at both residential and personal levels. Potential variation related to the use of multiple scores of the lymphocytes and the MN was reduced as a semi-automatic imaged based scoring system was developed and applied in the current study (Decordier et al. 2009; Vande Loock et al. 2011).

There were multiple potential sources of exposure classification error in our study. The models developed were based on a limited number of water analyses. The models assume that all of the homes within the same water system have the same level of exposures, and thus ignores potential spatial variation within the water systems. The addresses were generally from early pregnancy, and it was assumed that the mothers did not move later in their pregnancies. Self-reported estimates of water habits are likely to be prone to error. These errors are all expected to be non-differential with respect to MN formation. Under these conditions these errors generally, but not always, bias the exposure response toward the null (Armstrong 1998).

The estimated BTHM concentrations in residential water supplies for our study population were lower than concentrations reported for other populations. For example, the average residential THM concentration for a Spanish population was 33 μg/L (Villanueva et al. 2006), compared with a mean BTHM concentration of only 2 μg/L in our study. The residential drinking-water concentrations in our population were also much lower than the current permissible regulatory limits, which are 100 μg/L in the European Union and 80 μg/L in the United States.

In conclusion, we observed significant increases in the frequency of MN in maternal BN in association with exposure to BTHM in residential water during the first and second trimesters of pregnancy. We also observed an association between increased MN frequency in women who took baths, and a decreased frequency of MN among women who consumed primarily spring water, based on small numbers of observations. If these findings truly reflect a causal association, they may have important health and policy implications, particularly because the BTHM exposures in this study were considerably lower than the current standards limits for BTHM in drinking water.

## Supplemental Material

(205 KB) PDF
