# Erratum: “Exposure to Brominated Trihalomethanes in Water During Pregnancy and Micronuclei Frequency in Maternal and Cord Blood Lymphocytes”

**DOI:** 10.1289/ehp.122-A68

**Published:** 2014-03-01

In the article “Exposure to Brominated Trihalomethanes in Water During Pregnancy and Micronuclei Frequency in Maternal and Cord Blood Lymphocytes” by Stayner et al. [Environ Health Perspect 122:100–106 (2014)], there was an error in Table 1. The number of women in exposure quartile 4 who had missing data for maternal smoking was given as zero instead of 3. The corrected table appears below and has also been corrected online.

*EHP* regrets the error.

**Table 1:** Study population characteristics [*n* (%)]^*a*^ for all and by quartiles of the residential exposure to brominated trihalomethanes during the full pregnancy.^*b*^

Characteristic	All (*n* = 240)	Quartile 1	Quartile 2	Quartile 3	Quartile 4	*p*-Value^*c*^
Ethnicity						0.82
Greek	205 (86.3)	60 (88.2)	43 (84.3)	50 (83.3)	30 (88.1)
Other	33(13.9)	8 (11.8)	8 (15.7)	10 (16.7)	7 (11.9)
Missing	2	1	0	0	1
Maternal age (years)						0.24
≤ 35	210 (87.8)	65 (94.2)	44 (88.0)	50 (83.3)	51 (85.0)
> 35	29 (12.1)	4 (5.8)	6 (12.0)	10 (16.7)	9 (15.0)
Missing	1	0	1	0	0
Maternal smoking						0.06
Nonsmokers	145 (61.2)	45 (65.2)	33 (68.9)	28 (46.7)	39 (65.0)
Ex-smokers	44 (18.6)	16 (23.2)	5 (10.4)	14 (23.3)	9 (15.0)
Active smokers	48 (20.2)	8 (11.6)	10 (20.8)	18 (30.0)	12 (20.0)	
Missing	3	0	3	0	3
SHS^*d*^						0.42
Not exposed	26 (10.9)	5 (7.3)	5 (10.0)	6 (10.0)	10 (16.7)
Exposed	213 (89.1)	64 (92.8)	45 (90.0)	54 (90.0)	50 (83.3)
Missing	1	0	1	0	0
Maternal education						0.68
Low	55 (23.0)	19 (27.5)	11 (22.0)	13 (21.7)	12 (20.0)
Moderate	126 (52.7)	36 (52.2)	30 (60.0)	30 (50.0)	30 (50.0)
High	58 (24.3)	14 (20.3)	9 (18.0)	17 (28.3)	18 (30.0)
Missing	1	0	0	0	0
Residence						< 0.001
Urban	171 (72.1)	38 (55.9)	26 (52.0)	55 (91.7)	52 (88.1)
Rural	66 (27.9)	30 (44.1)	24 (48.0)	5 (8.3)	7 (11.9)
Missing	3	1	1	0	0
Gestational age (weeks)						0.98
≥37	225 (93.8)	64 (92.8)	48 (94.1)	57 (95.0)	56 (93.3)
< 37	15 (6.3)	5 (7.3)	3 (5.9)	3 (5.0)	4 (6.7)
Missing	0	0	0	0	0
Drinking water						< 0.001
Bottled	174 (72.8)	40 (58.0)	31 (62.0)	53 (88.3)	50 (83.3)
Public	49 (20.5)	23 (33.3)	15 (30.0)	6 (10.0)	5 (8.3)
Spring	16 (6.7)	6 (8.7)	4 (8.0)	1 (1.7)	5 (8.3)
Missing	1	0	1	0	0
Type of bathing						0.91
Shower only	221 (94.5)	63 (94.0)	48 (98.0)	55 (84.8)	55 (91.7)
Bath only	5 (2.1)	2 (3.0)	0 (0.0)	1 (1.7)	2 (3.3)
Both	8 (3.4)	2 (3.0)	1 (2.0)	2 (3.5)	3 (5.0)
Missing	6	2	2	2	0
Swimming pool use						0.24
Ever	229 (98.7)	67 (100.0)	47 (97.9)	57 (100.0)	58 (96.7)
Never	3 (1.3)	0 (0.0)	1 (2.1)	0 (0.0)	2 (3.3)
Missing	8	2	3	3	0
Hand dishwashing						0.21
No	82 (35.2)	25 (37.3)	13 (27.1)	26 (44.8)	18 (30.0)
Yes	151 (64.8)	42 (62.7)	35 (72.9)	32 (55.2)	42 (70.0)
Missing	7	2	3	2	0
All variables correspond to maternal characteristics during pregnancy. ^***a***^Number of subjects with micronuclei analyses (either mother or newborn). ^***b***^Brominated trihalomethanes in residential water quartiles: 0.06–0.15; > 0.15–0.74; > 0.74–4.44; > 4.44–7.09 μg/L. ^***c***^*p*-Value based on Fisher exact test for table ­frequencies (excluding the missing values). ^***d***^Exposed to secondhand smoke either at work or at home.						

