# Supplementary material for: Exposure to Brominated Trihalomethanes in Water During Pregnancy and Micronuclei Frequency in Maternal and Cord Blood Lymphocytes
Source: Environ Health Perspect. 2013 Nov 1;122(1):100–6. doi: 10.1289/ehp.1206434 (PMC3888564; doi:10.1289/ehp.1206434)
Supplement: (205 KB) PDF [file ehp.1206434.s001.508.pdf]

Supplemental Material

Exposure to Brominated Trihalomethanes in Water During Pregnancy and Micronuclei Frequency in Maternal and Cord Blood Lymphocytes

Leslie Thomas Stayner, Marie Pedersen, Evridiki Patelarou, Ilse Decordier, Kim Vande Loock, Leda Chatzi, Ana Espinosa, Eleni Fthenou, Mark J. Nieuwenhuijsen, Esther Gracia-Lavedan, Euripides G. Stephanou, Micheline Kirsch-Volders, and Manolis Kogevinas

Table of Contents

Table S1. Micronuclei frequency per 1000 binucleated or 1000 mononucleated cells mean (standard deviation) and p-values for trends by quartiles[a] of exposure to brominated trihalomethanes in residential water over the pregnancy……………………………………….2

Table S2. Associations between brominated trihalomethanes and micronuclei frequency in binucleated cells from cord blood (rate ratios and 95% CI)……………………….……………..3

Table S3: Associations between brominated trihalomethanes and micronuclei frequency in maternal mononucleated cells (rate ratios and 95% CI).…………………………...……….……4

Table S4: Associations between brominated trihalomethanes and micronuclei frequency in mononucleated cells from cord blood (rate ratios and 95% CI)………………………………..…5

Table S5. Associations between exposure to brominated trihalomethanes during the first trimester and micronuclei frequency in maternal binucleated cells from separate adjusted models stratified by maternal active tobacco smoking status (rate ratios and 95% CI)………………….. 6

Table S6. Associations between all routes of exposure to brominated trihalomethanes and micronuclei frequency in maternal binucleated cells from separate adjusted models stratified by maternal education (rate ratios and 95% CI)…………………………………………………..7

Figure S1: Estimated micronuclei frequency in maternal binucleated lymphocytes based on the unadjusted models for residential exposure to brominated thihalomethanes (μg/L)……..………8

Supplemental Material, Table S1. Micronuclei frequency per 1000 binucleated or 1000 mononucleated cells mean (standard deviation) and p-values for trends by quartiles[a] of exposure to brominated trihalomethanes in residential water over the pregnancy.

Group	Binucleated cells [b]	p [c]	Mononucleated cells [b]	p [c]
Mothers (N=189)[d]				
Quartile 1 (lowest, n=54)	2.68 ± 1.70		0.54 ± 0.79	
Quartile 2 (n=40)	2.34 ± 1.83		0.66 ± 1.02	
Quartile 3 (n=48)	2.96 ± 1.96		0.84 ± 1.32	
Quartile 4 (highest, n=47)	3.68 ± 3.02	0.07	0.89 ±1.13	0.02
Newborn children (N=195) [d]				
Quartile 1 (n=51)	2.05 ± 1.83		0.46 ± 0.45	
Quartile 2 (n=43)	1.52 ± 1.21		0.71 ± 0.91	
Quartile 3 (n=47)	2.07 ± 1.72		0.62 ± 0.71	
Quartile 4 (n=54)	1.71 ± 1.34	0.95	0.71 ± 0.77	0.25

[a] Brominated trihalomethanes in residential water quartiles: 0.06-≤ 0.15; >0 .15-≤ 0.74; >0.74-≤ 4.44; >4.44-7.09.

[b] The cells contain the arithmetic means ± standard deviations.

[c] The p-values are based on nonparametric test for trends across quartiles of exposures developed by Cuzick, which is an extension of the Wilcoxin rank-sum test

[d] The numbers of mothers and children in this analysis are less than the number used in the regression analyses. This is because this analysis was based on the unimputed dataset and the regression analyses were based on the imputed dataset. There were 25 mothers and 28 children in the unimputed dataset who were missing BTHM data who were included in the regression analysis.

Supplemental Material, Table S2. Associations between brominated trihalomethanes and micronuclei frequency in binucleated cells from cord blood (rate ratios and 95% CI).

Exposure	Unadjusted	Adjusted[a]
Residential water (µg/L)		
1st Trimester	1.01 (0.95, 1.07)	1.02 (0.96, 1.09)
2nd Trimester	0.99 (0.96, 1.02)	0.99 (0.96, 1.02)
3rd Trimester	0.98 (0.96, 1.01)	0.98 (0.95, 1.01)
Full Pregnancy	0.98 (0.95, 1.03)	0.99 (0.94, 1.03)
All routes of exposure (µg/week)		
1st Trimester	0.74 (0.18, 3.04)	1.02 (0.25, 4.26)
2nd Trimester	0.61 (0.28, 1.33)	0.64 (0.30, 1.39)
3rd Trimester	0.71 (0.32, 1.55)	0.63 (0.30, 1.34)
Full Pregnancy	0.58 (0.21, 1.62)	0.59 (0.22, 1.58)

Rate ratios and 95%CI of the micronuclei frequency in maternal mononucleated cells from negative binomial models (per 1 unit of exposure increment).

[a]Adjusted for gestational age, maternal tobacco smoking, maternal exposure to workplace and home second-hand smoke, living in an urban area, maternal education and Greek ethnicity.

Supplemental Material, Table S3: Associations between brominated trihalomethanes and micronuclei frequency in maternal mononucleated cells (rate ratios and 95% CI).

Exposure	Unadjusted	Adjusted[a]
Residential water (μg/L)		
1st Trimester	1.05 (0.99, 1.11)	1.00 (0.92, 1.08)
2nd Trimester	1.04 (1.00, 1.09)	1.02 (0.98, 1.07)
3rd Trimester	1.01 (0.96, 1.06)	1.02 (0.97, 1.06)
Full Pregnancy	1.04 (0.97, 1.11)	1.02 (0.96, 1.08)
All routes of exposure (μg/week)		
1st Trimester	2.22 (0.69, 7.41)	0.91 (0.17, 4.99)
2nd Trimester	2.36 (0.83, 6.75)	1.87 (0.55, 6.40)
3rd Trimester	0.62 (0.39, 1.02)	1.61 (0.47, 5.50)
Full Pregnancy	2.02 (0.52, 7.90)	1.84 (0.37, 8.98)

Rate ratios and 95%CI of the micronuclei frequency in maternal mononucleated cells from negative binomial models (per 1 unit of exposure increment).

[a]Adjusted for maternal age at birth, maternal tobacco smoking, maternal exposure to workplace and home second-hand smoke, living in an urban area, maternal education and Greek ethnicity.

Supplemental Material, Table S4. Associations between brominated trihalomethanes and micronuclei frequency in mononucleated cells from cord blood (rate ratios and 95% CI).

Exposure	Unadjusted	Adjusted[a]
Residential water (μg/L)		
1st Trimester	1.01 (0.95, 1.07)	1.00 (0.93, 5.73)
2nd Trimester	1.03 (0.99, 1.06)	1.03 (0.98, 1.07)
3rd Trimester	1.02 (0.98, 1.06)	1.02 (0.97, 1.07)
Full Pregnancy	1.03 (0.97, 1.08)	1.03 (0.97, 1.09)
All routes of exposure (μg/week)		
1st Trimester	1.11 (0.25, 5.10)	1.04 (0.19, 5.73)
2nd Trimester	1.97 (0.68, 5.68)	2.06 (0.62, 6.85)
3rd Trimester	1.77 (0.57, 5.47)	1.74 (0.51, 5.97)
Full Pregnancy	2.06 (0.52, 8.30)	2.07 (0.43, 9.99)

Rate ratios and 95%CI of the micronuclei frequency in maternal mononucleated cells from negative binomial models (per 1 unit of exposure increment).

[a]Adjusted for gestational age, maternal tobacco smoking, maternal exposure to workplace and home second-hand smoke, living in an urban area, maternal education and Greek ethnicity.

Supplemental Material, Table S5. Associations between exposure to brominated trihalomethanes during the first trimester and micronuclei frequency in maternal binucleated cells from separate adjusted models stratified by maternal active tobacco smoking status (rate ratios and 95% CI) [a].

Smoking status	Residential Water (µg/L)	All Routes of Exposure (µg/week)
Nonsmokers (n=145)	0.99 (0.93, 1.04)	1.23 (0.34, 4.50)
Exsmokers (n=44)	1.18 (0.99, 1.42)	0.18 (0.10, 2.48)
Active Smokers (n=48)	1.11 (1.04, 1.18)	3.97 (0.88, 17.99)

[a]Rate ratios and 95%CI of the micronuclei frequency in maternal binucleated cells from negative binomial models (per 1 unit of exposure increment) adjusted for maternal age at birth, maternal exposure to workplace and home second-hand smoke, living in an urban area, maternal education and Greek ethnicity.

[b]Nonsmokers did not report smoking at any time for at least 3 months prior to pregnancy or during pregnancy; Exsmokers reported smoking sometime within the 3 months prior to pregnancy and/or during the first 12 weeks of gestation, but not after then, and; Active smokers reported smoking 3 months prior to pregnancy and at approximately 12 weeks of gestation (the time of interview).

Supplemental Material, Table S6. Associations between all routes of exposure to brominated trihalomethanes and micronuclei frequency in maternal binucleated cells from separate adjusted models stratified by maternal education (rate ratios and 95% CI)[a].

All routes of exposure (μg/week)	Level of Education [b]		
	Low (n=47)	Medium (n=114)	High (n=44)
1st Trimester	18.09 (1.31, 250.04)	1.75 (0.65, 4.74)	0.26 (0.00, 15.90)
2nd Trimester	7.98 (1.71, 37.17)	0.74 (0.33, 1.66)	0.54 (0.13, 2.23)
3rd Trimester	1.59 (0.55, 4.63)	0.47 (0.22, 1.03)	0.52 (0.18, 1.54)
Full Pregnancy	10.88 (1.70, 69.48)	0.71 (0.23, 2.19)	0.39 (0.06, 2.40)

[a]Rate ratios and 95%CI of the micronuclei frequency in maternal mononucleated cells from negative binomial models (per 1 unit of exposure increment) adjusted for maternal age at birth, maternal exposure to workplace and home second-hand smoke, living in an urban area, maternal education and Greek ethnicity.

[b] Low level: ≤6 years of school, medium level: < 12 years of school, high level: university or technical college degree)

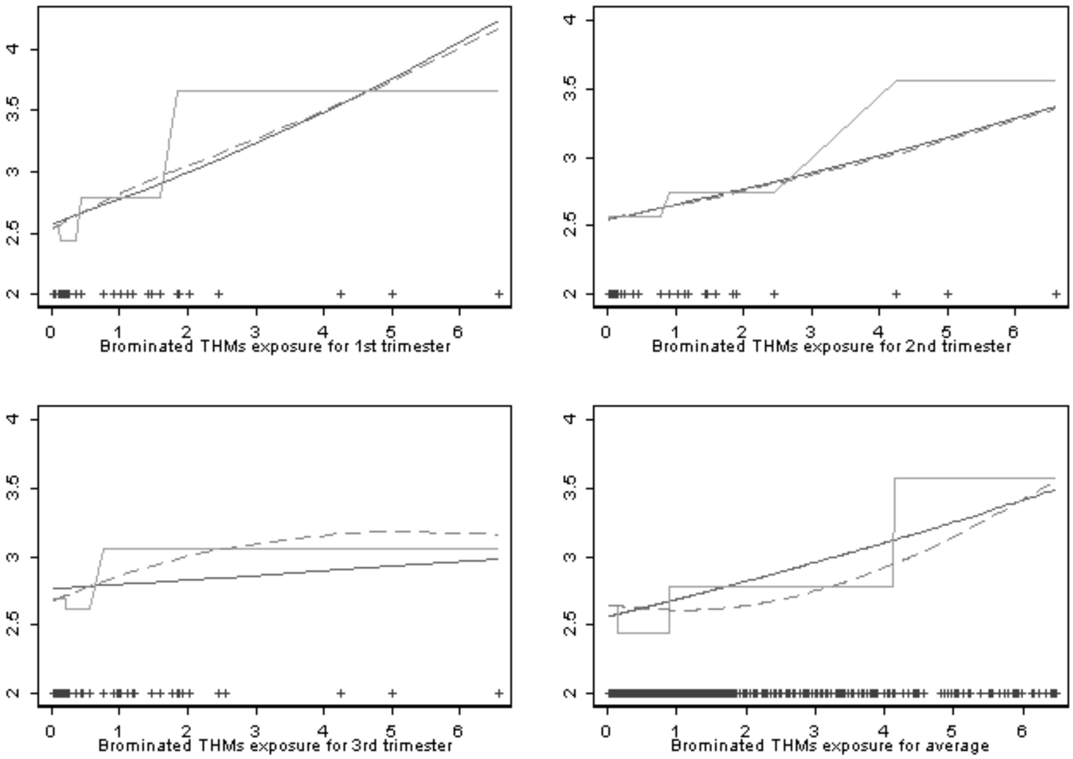

Supplemental Material, Figure S1. Estimated micronuclei frequency in maternal binucleated lymphocytes based on the unadjusted models for residential exposure to brominated trihalomethanes (μg/L). Results for continuous exposure (solid curve), categorical exposure (step function) and cubic spline exposure-response models (dashed curve). Scatter points at the bottom are the observed values of the exposure. Results plotted by trimester-specific exposures and exposure averaged over the full pregnancy.
